# International survey on the use of tranexamic acid in plastic surgery—Current practices and perspectives

**DOI:** 10.1016/j.jpra.2026.03.031

**Published:** 2026-04-03

**Authors:** Irene Mesas Aranda, Benedikt Fuchs, Felix H. Vollbach, Nikolaus Thierfelder, Riccardo E. Giunta, Konstantin Koban, Nicholas Moellhoff, Sinan Mert

**Affiliations:** a Division of Hand, Plastic and Aesthetic Surgery, LMU University Hospital, Munich, Germany; b Department of Plastic, Reconstructive and Aesthetic Surgery, University Medical Center Hamburg-Eppendorf, Hamburg, Germany

**Keywords:** Tranexamic acid, Plastic surgery, Bleeding, International survey

## Abstract

**Background:**

Tranexamic acid (TXA) has gained increasing popularity in plastic and reconstructive surgery for minimizing perioperative bleeding and improving postoperative outcomes. However, standardized guidelines on its use remain lacking. Methods: An anonymous international survey was distributed to over 400 plastic surgeons worldwide to evaluate current TXA usage patterns, including dosage, timing, route of administration as well as perceived efficacy and safety.

**Results:**

Sixty-nine fully completed responses from 17 countries were analyzed. Overall, 86.9% of respondents reported TXA use, primarily to reduce blood loss and postoperative bruising. Intravenous administration was preferred by 55.9%, topical by 15.3%, and 28.8% used both. The most common intravenous dose was 10–14 mg/kg BW, usually given intraoperatively or within 30 min before incision. Topical TXA was typically applied during hemostasis or wound closure, most frequently undiluted (100 mg/mL) or in diluted solutions (10–50 mg/mL). Use was highest in aesthetic bodycontouring and breast procedures but remained low in microvascular and burn surgery. Almost all respondents (98%) reported no TXA-related complications. No thromboembolic or neurological adverse events occurred.

**Conclusions:**

TXA is widely and safely implemented in plastic surgery, particularly in aesthetic procedures, but substantial heterogeneity exists regarding dosage, timing, and route of application. These findings underscore the need for procedure-specific, evidence-based protocols and prospective multicenter trials to standardize TXA use in plastic and reconstructive surgery.

## Introduction

Tranexamic acid (TXA) is an antifibrinolytic agent, first discovered in the early 1960s by Utako Okamoto.^1^ Its mechanism of action involves competitive inhibition of the lysine-binding sites on plasminogen, thereby preventing its activation by plasminogen activators (e.g., tissue plasminogen activator, t-PA). This results in the attenuation of hyperfibrinolysis and contributes to the stabilization of formed blood clots.^2^ Initially developed for the management of peripartum hemorrhage, TXA has since become an integral therapeutic component across a wide range of medical disciplines, including gynecology, cardiovascular surgery, traumatology, and orthopedics. Several studies have already demonstrated that TXA significantly reduces perioperative blood loss and the need for blood transfusions.^3^, ^4^, ^5^ Nevertheless, the use of TXA is predominantly off-label, as its official indication according to the European Medicines Agency (EMA) is limited to prevention and treatment of bleeding resulting from localized or generalized hyperfibrinolysis in adults and children above one year of age.^6^ The only FDA-approved indications for use of TXA are heavy menstrual bleeding and short-term prevention of bleeding in patients with hemophilia.^7^

In recent years, the use of TXA has gained tremendous interest in plastic, reconstructive and aesthetic surgery. A targeted PubMed search using the query “tranexamic acid” and “plastic surgery” identified approximately 250 publications, with nearly 150 of these published within the last three years. However, <10% of these studies constitute randomized controlled trials, highlighting a notable gap in high-quality evidence. The most commonly reported indications include breast surgery, postbariatric procedures, cosmetic interventions and burn surgery.^8^ This growing interest is not coincidental. Plastic and reconstructive surgery poses unique hemostatic challenges as procedures frequently involve large skin flaps, extensive subcutaneous dissection, and wide operative fields predisposing to diffuse microvascular blood loss, where even minor postoperative hematoma can have important consequences. In body-contouring surgery, abdominoplasty carries the highest rates of readmission and revision surgery.^9^ In implant-based breast augmentation or reconstruction, hematoma is a well-established trigger of capsular contracture, occurring in 25% of affected patients compared to only 8% in those without.^10^ In free-flap microsurgery, perivascular bleeding may compromise flap viability. With this background, TXA has attracted growing interest: a meta-analysis of body-contouring procedures demonstrated a significant reduction in hematoma risk with TXA,^11^ and studies in abdominoplasty have shown significant reductions in seroma formation following intravenous administration.^12^ Effective hemostasis thus directly impacts patient safety, wound healing, and surgical outcome.

Despite its widespread clinical use, standardized and evidence-based recommendations for TXA administration in plastic surgery remain absent. The aim of the present survey is therefore to provide a comprehensive overview of current TXA practices among plastic surgeons worldwide, to characterize regional variations in its application, and to develop practical recommendations for its use in clinical practice.

## Methods

To analyze current trends in the use of tranexamic acid to reduce peri‑ and postoperative bleeding and bleeding complications, an online survey was designed with the online survey tool LimeSurvey (LimeSurvey GmbH, Germany). The survey was conducted completely anonymously in accordance with the General Data Protection Regulation of the European Union. To maximize international participation, the questionnaire was designed in English and sent to over 400 plastic surgeons from all over the world.

The survey consisted of 34 questions, divided into 5 subgroups. The complete questionnaire can be found in the supplementary data.

a) Demographics
b) General questions regarding the use of tranexamic acid
c) Intravenous application of tranexamic acid
d) Topical application of tranexamic acid
e) Further questions

Plastic surgeons were contacted by e-mail correspondence through contact listings in national and international specialty societies. No reminders were sent. The survey was conducted from July 2024 to June 2025. Only fully completed questionnaires were included in the final analysis. The responses were tabulated, and statistical analyses were performed using SPSS 21 (IBM, United States). Descriptive statistics were used to summarize categorical and ordinal data. Associations between categorical variables were assessed using the Chi-square test or Fisher–Freeman–Halton exact test, as appropriate. Effect sizes were calculated using Cramer’s V. Statistical significance was set at *p* < 0.05.

## Results

### Respondent profile

**Figure 1:**
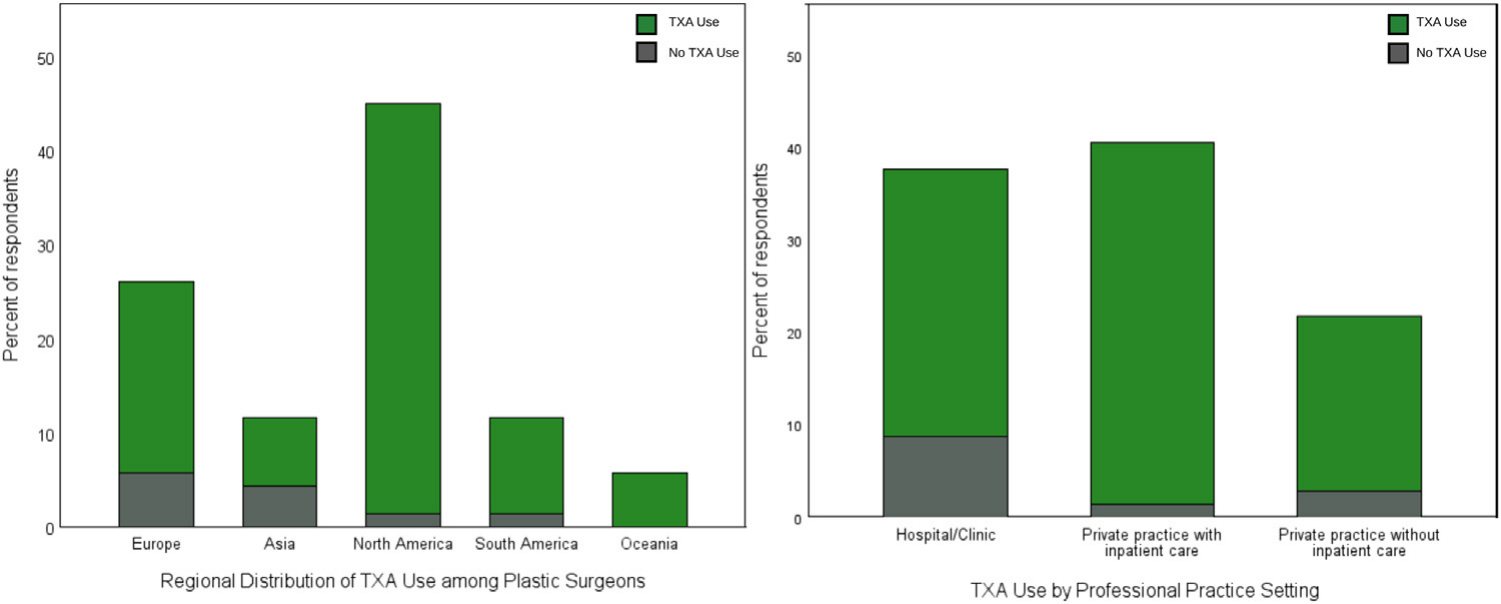
Geographical distribution of tranexamic acid use and its relationship to professional practice setting among surveyed plastic surgeons. Left panel: Stacked bar chart depicting the proportion of respondents reporting TXA use (green) versus non-use (grey) across five geographic regions. Right panel: TXA use stratified by professional practice setting. TXA, tranexamic acid.

A total of 71 of 423 invited plastic surgeons completed the questionnaire, corresponding to a response rate of 16.8%. Two incomplete or non-displayed responses were excluded, leaving 69 valid datasets for analysis. Datasets from 17 countries across five continents were analyzed, with most practicing in North America (44.9%; Figure 1A).

Most respondents worked in private practice with inpatient care or hospital settings (Figure 1B). Almost half primarily performed aesthetic surgery (47.9%), while 31.0% focused on reconstructive procedures and 21.1% on both.

### General use of tranexamic acid

**Figure 2:**
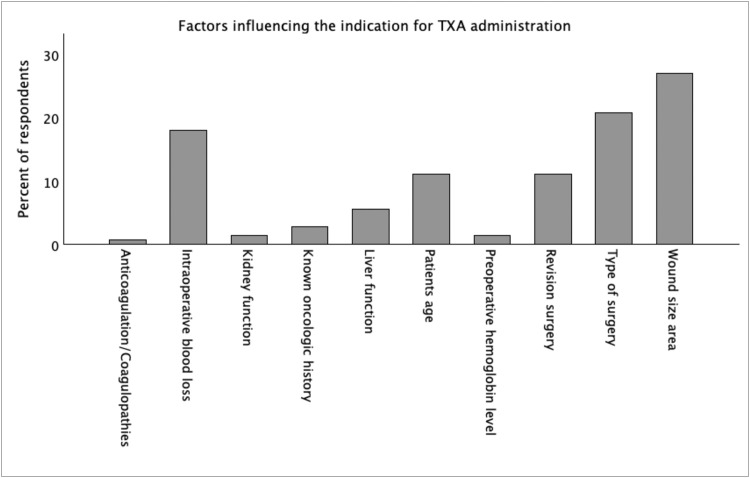
Clinical factors cited as determinants for tranexamic acid administration in plastic surgery. Bar chart displaying the percentage of respondents identifying each parameter as relevant to their indication for TXA use. Wound size area and type of surgery were the most frequently cited criteria. TXA, tranexamic acid.

Overall, 86.9% of participants reported routine TXA use, with the highest adoption observed in North America (Figure 1). TXA use was significantly higher in the America than in Europe or Asia–Oceania (94.9% vs 76.7%; Fisher’s exact *p* = 0.035). TXA administration was primarily driven by procedural rather than patient-specific factors. Surgeons based their decision mainly on the type of surgery, wound size, and anticipated intraoperative blood loss, whereas laboratory values and comorbidities had minimal influence on clinical decision-making (Figure 2). Respondents who did not routinely use TXA reported relying on conventional hemostatic strategies first. These primarily included epinephrine-containing infiltration and postoperative compression, complemented by intraoperative blood pressure elevation during hemostasis and occasional use of topical hemostatic agents. Additional measures such as meticulous cautery-based hemostasis and preoperative bleeding-risk assessment were also mentioned.

Most respondents had several years of experience using TXA and considered it effective for reducing perioperative bleeding (92.7%). Perceived usefulness was consistent across practice settings and administration routes. Agreement with anesthesiologists was high (89.0%) and adverse events were very rare, with no reported thromboembolic event and only a single allergic reaction.

**Figure 3:**
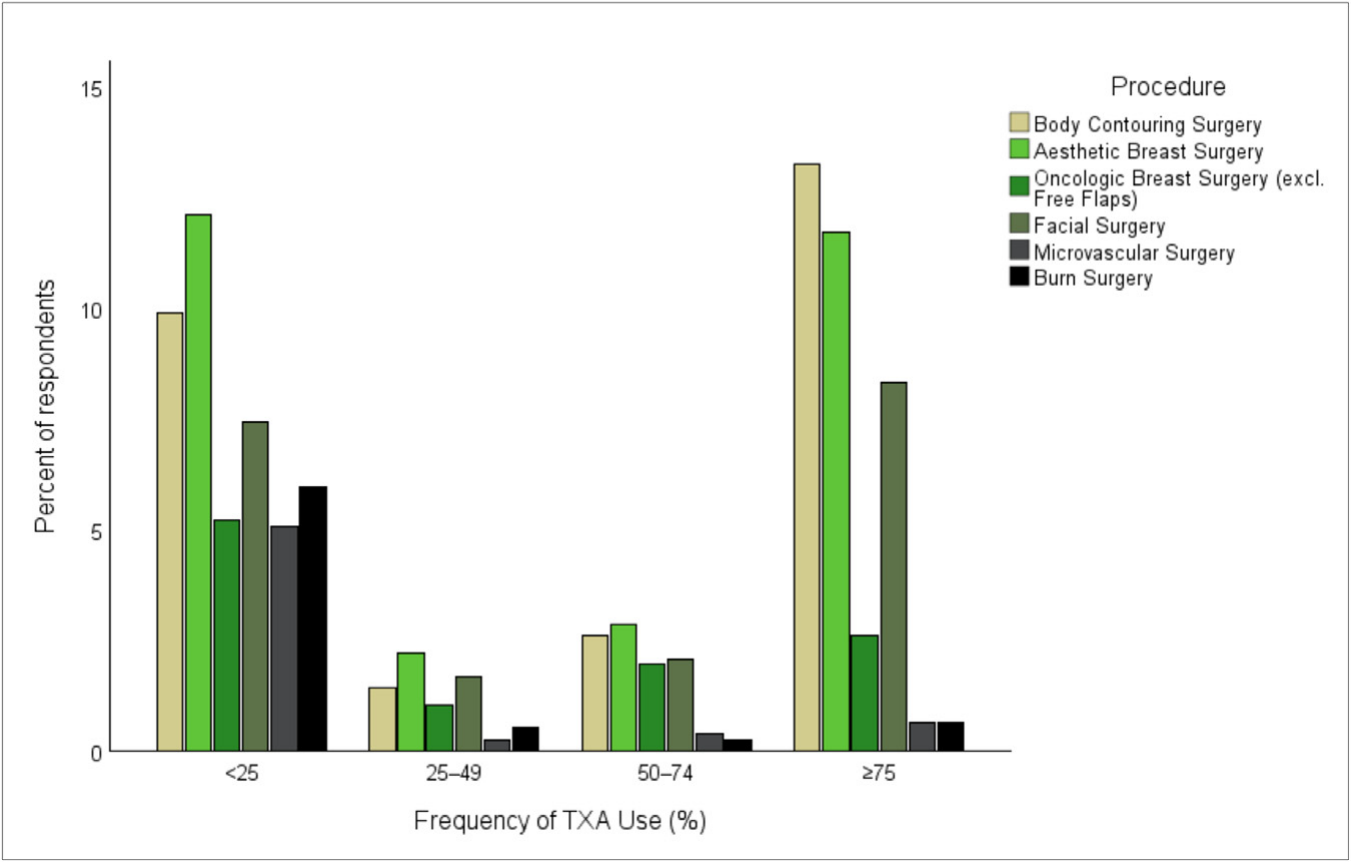
Self-reported frequency of tranexamic acid use depending on surgical subspecialty. Grouped bar chart showing the percentage of respondents reporting TXA application across four frequency categories (<25%, 25%–49%, 50%–74%, and ≥75% of eligible cases) for six surgical subspecialties. TXA, tranexamic acid.

TXA utilization varied markedly across procedures. High-frequency use (≥75.0%) clustered in aesthetic surgery, particularly bodycontouring procedures and breast surgery, whereas it was substantially lower in microvascular and burn surgery (Figure 3). Overall, use decreased along the spectrum from aesthetic to reconstructive procedures (χ² = 125.8, *p* < 0.001) and was independent of operative case volume.

### Intravenous administration of TXA

**Figure 4:**
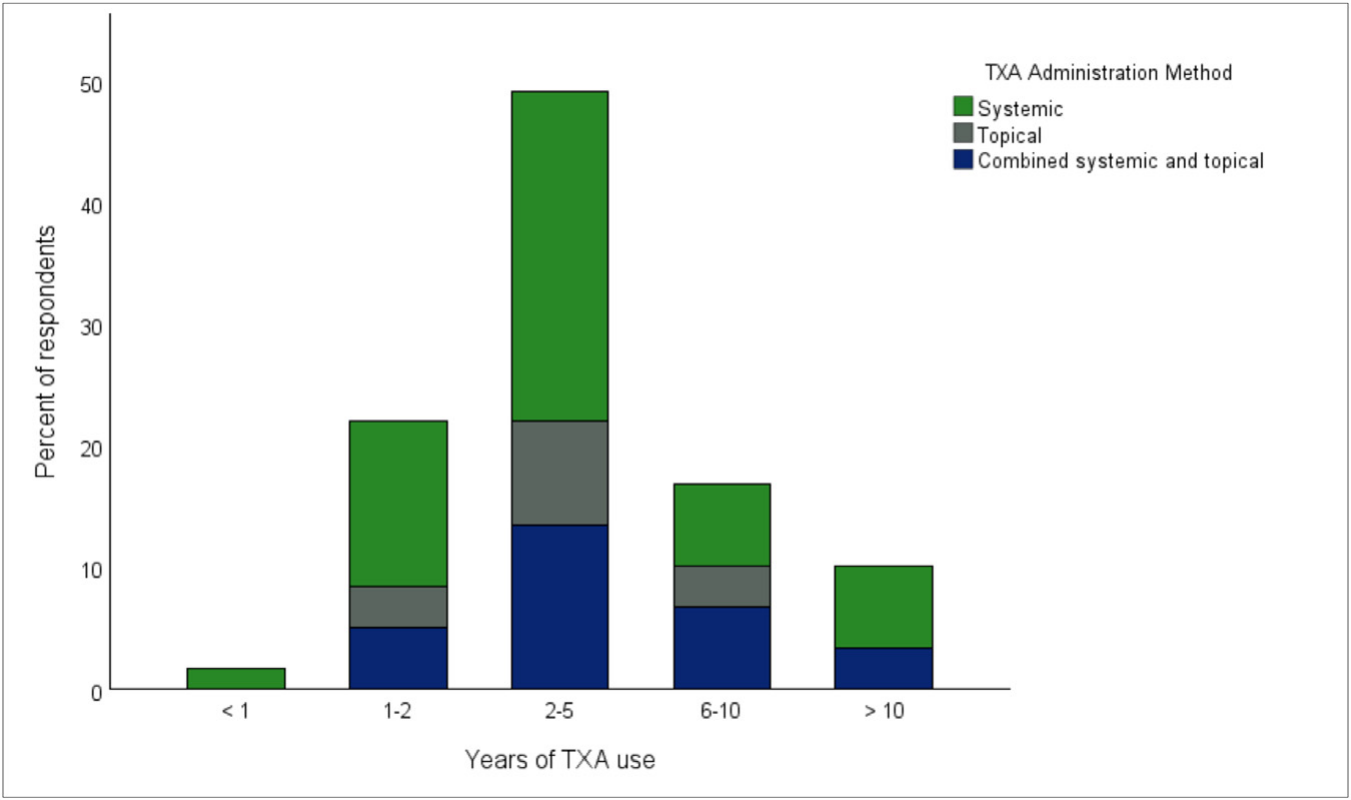
Route of tranexamic acid administration stratified by duration of clinical experience with TXA. Stacked bar chart illustrating the proportion of respondents employing systemic (green), topical (grey), or combined systemic and topical (blue) TXA administration, categorized by years of TXA experience (<1, 1–2, 2–5, 6–10, and >10 years). TXA, tranexamic acid.

Among TXA users, 55.9% preferred intravenous administration, 15.3% topical only, and 28.8% a combined application. Administration route was independent of region, practice setting and years of experience (Figure 4).

Intravenous TXA was primarily used in aesthetic and body-contouring procedures, while use remained limited in reconstructive, oncologic, microvascular, and burn surgery.

**Figure 5:**
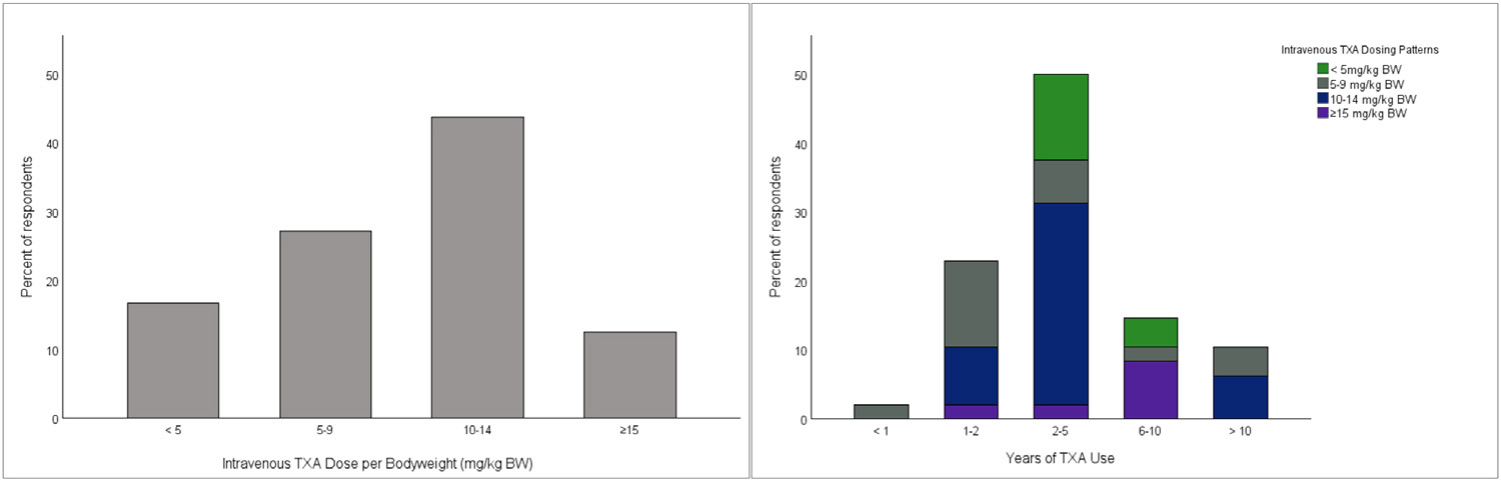
Intravenous tranexamic acid dosing practices and their relationship to clinical experience in plastic surgery. Left panel: Distribution of intravenous TXA dose per kilogram of body weight (mg/kg BW) among all respondents. Right panel: Stacked bar chart stratifying intravenous TXA dosing patterns (<5, 5–9, 10–14, and ≥15 mg/kg BW) by years of clinical TXA experience. BW, body weight; TXA, tranexamic acid.

The most common dose was 10–14 mg/kg body weight (BW) (Figure 5A), typically administered as a single intraoperative bolus or short infusion, most often at the time of hemostasis (34.7%), within 30 min before incision (24.0%), or at incision (17.3%). A significant correlation was found between years of TXA use and the intravenous dosing pattern (χ² = 30.76, *p* = 0.01; Figure 5B), indicating that more experienced users tended to apply doses of 10–14 mg/kg BW.

Repeated dosing was uncommon and generally reserved for high-risk situations, mainly guided by intraoperative or postoperative bleeding. When repeated, administration was generally limited to 24–72 h, at 6–8-hour intervals.

Only 1.4% of participants reported allergic reactions. No seizures, thromboembolic, or visual complications were reported.

### Topical application

**Figure 6:**
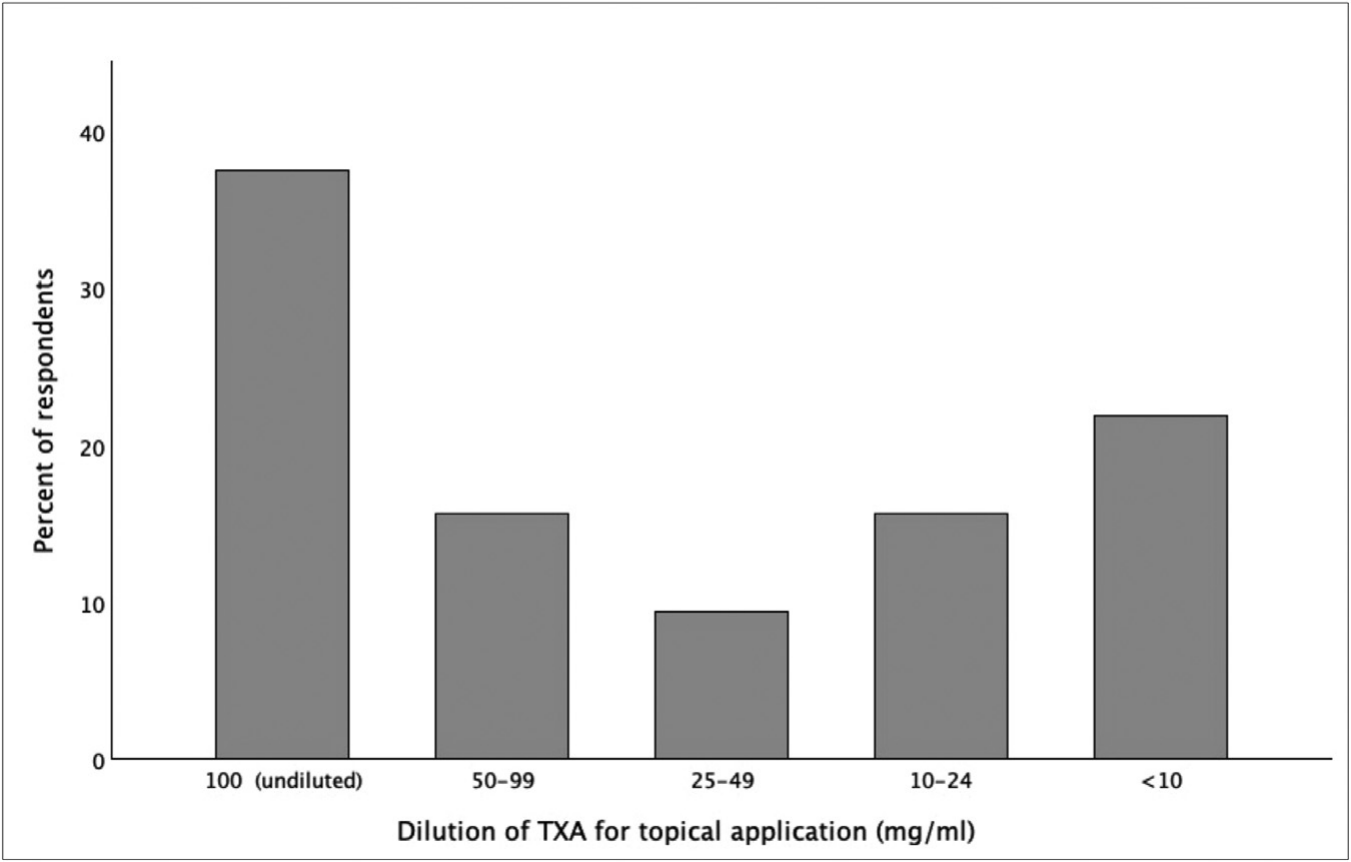
Concentration of tranexamic acid used for topical application in plastic surgery. Bar chart illustrating the distribution of TXA dilutions used for topical administration across the surveyed population, ranging from undiluted commercial solution (100 mg/mL) to highly diluted preparations (<10 mg/mL). TXA, tranexamic acid.

Topical TXA was used by 44.1% of respondents, either alone or combined with intravenous administration. The most common concentration was undiluted 100 mg/mL (37.5%; Figure 6), applied via irrigation or soaked gauze during hemostasis or before wound closure. TXA was generally not added to the tumescent solution during liposuction procedures (64.4%). When used, the most common choices were 500–990 mg/L (15.5%) and 100–240 mg/L (13.3%).

Topical TXA was primarily applied in aesthetic procedures, particularly facial and breast surgery, and was rarely used in reconstructive, microvascular, or burn surgery. Compared with intravenous use, topical TXA was less common but associated with subjectively reduced bruising, edema, and drain output, especially in facial and breast surgery. No topical-specific adverse events were reported.

## Discussion

The present international survey provides a global overview of current practices regarding the use of tranexamic acid (TXA) among plastic surgeons. The findings confirm that TXA has been widely integrated into daily surgical practice, particularly in body-contouring and aesthetic procedures, while its use in reconstructive and microvascular surgery remains less common. Despite this broad acceptance, considerable variability persists in dosing, timing, and route of administration, underscoring the lack of standardized, evidence-based protocols in the use of TXA in plastic surgery.

Regarding the administration method, TXA is most commonly applied intravenously (84.7% of the users). Most surgeons administered a single intraoperative bolus of approximately 10–14 mg/kg BW, typically within 30 min of incision or at the time of hemostasis. Topical TXA was applied mainly via irrigation or soaked gauze.

Several meta-analyses and randomized studies have consistently demonstrated that both intravenous and local TXA significantly reduce intraoperative blood loss and postoperative hematoma formation without increasing thromboembolic risk.^11^^,^^13^^,^^14^

In fact, a large number of studies use weight-adapted, intravenous regimens of around 10–15 mg/kg BW or fixed doses of 1–2 g, which have shown comparable hemostatic effects.^11^^,^^13^^,^^15^, ^16^, ^17^, ^18^

Across multiple systematic reviews and meta-analyses no consistent advantage of repeated dosing has been shown,^11^^,^^13^, ^14^, ^15^, ^16^^,^^19^, ^20^, ^21^ and only very few studies reported a clear advantage of repeated administration.^22^^,^^23^ Consistent with our study findings, almost 63% of respondents preferred a single administration.

Topical application was most often used undiluted (100 mg/mL) or at concentrations between 50 and 99 mg/mL, depending on the procedure and institution. In the literature, concentrations of 20–25 mg/mL (using 20–25 mL) appear effective for achieving hemostasis and reducing drain output, especially in breast and facial surgery.^15^^,^^19^^,^^24^ Less diluted preparations (50 mg/mL) were mainly used for irrigation during body-contouring procedures,^25^ whereas tumescent solutions for liposuction often contained 0.5–1 g TXA per liter of tumescence solution.^14^ These values correspond closely to those cited by our respondents.

The question of whether intravenous or topical TXA is superior remains unresolved. Owing to their stronger local effect and lower systemic concentrations,^18^ Isaev et al.^15^ argued in favor of topical application in plastic surgery. In contrast, several other studies^11^^,^^13^^,^^16^ reported a more pronounced hemostatic effect with intravenous administration. Importantly, all studies regardless of route, found TXA to be safe within the dosing ranges described above, consistent with the very low complication rates reported by our respondents. Adverse events were reported only at supra-therapeutic doses (>80 mg/kg BW^26^) or high topical doses.^27^ In our survey, over 80% of respondents had never observed a TXA-related complication, and no thromboembolic or seizure events were reported. Given the self-reported, cross-sectional nature of the data, however, these findings should be interpreted with caution, as recall bias and underreporting are inherent limitations of survey methodology. Nonetheless, the results are consistent with the existing literature and support the view that TXA, used within standard dosing ranges, carries a low complication risk in plastic surgery, a premise that prospective studies should formally validate.^11^^,^^15^^,^^16^^,^^24^

In line with our results, TXA use was most prevalent in aesthetic procedures, particularly in body-contouring and large-volume liposuction. In breast surgery, TXA was widely used, especially in reduction mammaplasties.^28^ Alhebshi et al.^24^ and Pontes et al.^17^ demonstrated significant reductions in postoperative bleeding and drainage at topical concentrations of 20–25 mg/mL (using 20–25 mL)typically applied after hemostasis and before closure—mirroring respondent practice.

In facial aesthetic surgery, topical TXA significantly reduces ecchymosis and improves visualization.^19^^,^^29^ No thromboembolic or wound-healing complications have been reported to date, supporting the excellent safety profile of topical TXA in this field.

In contrast, reconstructive breast surgery showed lower TXA utilization in our survey. Evidence remains mixed. A multicenter analysis of >3800 implant-based reconstructions found no significant reduction in hematoma or seroma formation following a single 1 g intravenous application.^30^ For free-flap surgery, only small retrospective series exist. While none reported vascular compromise, a clear hemostatic benefit has yet to be demonstrated.^31^^,^^32^ The reassuring safety data suggest that future randomized controlled trials could investigate TXA’s role in microsurgery.

Finally, TXA was rarely employed in burn surgery in our survey. Meta-analyses^33^^,^^34^ indicate that intravenous TXA can significantly lower intraoperative blood loss and transfusion requirements without increasing thromboembolic or seizure events. Despite good safety and efficacy, routine implementation remains low, and standardized protocols are needed.

This survey delineates global practice patterns for TXA use exclusively among plastic surgeons. Limitations include a modest response rate (16.8%), uneven geographical representation with overrepresentation of North America, and a sample rate that limits statistical power for subgroup analyses. Furthermore, the self-reported nature of the data introduces potential recall bias, and the absence of reported complications cannot be equated with verified safety outcomes

Overall, current practice patterns broadly align with the published evidence: TXA provides hemostatic benefit in body-contouring and facial procedures as well as breast, and an emerging yet underused potential in burn and microsurgical procedures. The development of procedure-specific, evidence-based guidelines would enhance consistency and help optimize TXA application across domains of plastic surgery.

## Conclusion

This international survey demonstrates that TXA has been broadly adopted in plastic surgery, particularly for aesthetic body-contouring and breast procedures, with a single intravenous dose of 10–14 mg/kg BW as the most common approach. Regional differences in adoption were observed, and the safety profile was favorable. Despite widespread use, considerable heterogeneity persists in dosing, timing, and route of administration, highlighting the need for procedure-specific, evidence-based protocols. Prospective multicenter trials are warranted, particularly in microsurgery and burn surgery where evidence remains limited.

## Funding

This research did not receive any specific grant from funding agencies in the public, commercial, or not-for-profit sectors.

## Declaration of competing interest

None.

## Appendix Supplementary materials

Image, application 1
