# Supplementary material for: International survey on the use of tranexamic acid in plastic surgery—Current practices and perspectives
Source: JPRAS Open. 2026 Apr 3;50:1–9. doi: 10.1016/j.jpra.2026.03.031 (PMC13126280; doi:10.1016/j.jpra.2026.03.031)
Supplement: Supplementary file 1 [file mmc1.pdf]

Survey - Use of Tranexamic Acid to reduce peri- and postoperative bleeding and bleeding complications in Plastic Surgery

Dear colleagues,

tranexamic acid offers enormous potential for reducing peri- and postoperative bleeding and bleeding complications, thereby enhancing patient safety and satisfaction. Despite its growing popularitiy, standardised guidelines for its use in plastic surgery have not yet been established.

With this survey, we aim to gain a comprehensive insight into the use of tranexamic acid in plastic surgery and to develop consensus recommendations.

Thank you for your participation in our survey.

Division of Hand, Plastic and Aesthetic Surgery

LMU University Hospital Munich

There are 34 questions in this survey.

Demographics

What is your professional setting? *

❗ Choose one of the following answers
Please choose **only one** of the following:

◯ Hospital/Clinic

◯ Private practice with inpatient care

◯ Private practice without inpatient care

In which country do you work? *

❗ Choose one of the following answers
Please choose **only one** of the following:

- ◯ Afghanistan
- ◯ Albania
- ◯ Algeria
- ◯ Andorra
- ◯ Angola
- ◯ Argentina
- ◯ Armenia
- ◯ Australia
- ◯ Austria
- ◯ Azerbaijan
- ◯ Bahamas
- ◯ Bahrain
- ◯ Bangladesh
- ◯ Belarus
- ◯ Belgium
- ◯ Bolivia
- ◯ Bosnia Herzegovina
- ◯ Brazil
- ◯ Bulgaria
- ◯ Cambodia
- ◯ Cameroon
- ◯ Canada
- ◯ Central African Republic
- ◯ Chile
- ◯ China
- ◯ Colombia

- ◯ Congo
- ◯ Costa Rica
- ◯ Croatia
- ◯ Cuba
- ◯ Cyprus
- ◯ Czech Republic
- ◯ Denmark
- ◯ Dominican Republic
- ◯ Ecuador
- ◯ Egypt
- ◯ El Salvador
- ◯ Eritrea
- ◯ Estonia
- ◯ Ethiopia
- ◯ Fiji
- ◯ Finland
- ◯ France
- ◯ Gabon
- ◯ Gambia
- ◯ Georgia
- ◯ Germany
- ◯ Ghana
- ◯ Greece
- ◯ Guatemala
- ◯ Guinea
- ◯ Haiti
- ◯ Honduras
- ◯ Hungary
- ◯ Iceland
- ◯ India
- ◯ Indonesia

- Iran
- Iraq
- Ireland
- Israel
- Italy
- Jamaica
- Japan
- Jordan
- Kazakhstan
- Kenya
- Korea, North
- Korea, South
- Kosovo
- Kuwait
- Laos
- Latvia
- Lebanon
- Lesotho
- Liberia
- Libya
- Liechtenstein
- Lithuania
- Luxembourg
- Madagascar
- Malaysia
- Maldives
- Mali
- Malta
- Mauritius
- Mexico
- Moldova

- ◯ Monaco
- ◯ Mongolia
- ◯ Montenegro
- ◯ Morocco
- ◯ Myanmar
- ◯ Namibia
- ◯ Nepal
- ◯ Netherlands
- ◯ New Zealand
- ◯ Nicaragua
- ◯ Niger
- ◯ Nigeria
- ◯ North Macedonia
- ◯ Norway
- ◯ Oman
- ◯ Pakistan
- ◯ Panama
- ◯ Paraguay
- ◯ Peru
- ◯ Philippines
- ◯ Poland
- ◯ Portugal
- ◯ Qatar
- ◯ Romania
- ◯ Russia
- ◯ Rwanda
- ◯ Samoa
- ◯ San Marino
- ◯ Saudi Arabia
- ◯ Senegal
- ◯ Serbia

- ◯ Singapore
- ◯ Slovakia
- ◯ Slovenia
- ◯ Somalia
- ◯ South Africa
- ◯ Spain
- ◯ Sri Lanka
- ◯ Sudan
- ◯ Suriname
- ◯ Sweden
- ◯ Switzerland
- ◯ Syria
- ◯ Taiwan
- ◯ Thailand
- ◯ Trinidad and Tobago
- ◯ Tunisia
- ◯ Turkey
- ◯ Turkmenistan
- ◯ Uganda
- ◯ Ukraine
- ◯ United Arab Emirates
- ◯ United Kingdom
- ◯ United States
- ◯ Uruguay
- ◯ Uzbekistan
- ◯ Venezuela
- ◯ Vietnam
- ◯ Yemen
- ◯ Zambia
- ◯ Zimbabwe

How many of the following procedures do you perform **monthly**? *

Please choose the appropriate response for each item:

	None	1-5 monthly	6-10 monthly	11-20 monthly	> 20 monthly
**Body-contouring procedures (excluding breast surgery)**	○	○	○	○	○
**Breast surgery procedures (excluding oncological procedures)**	○	○	○	○	○
**Oncological breast surgery procedures**	○	○	○	○	○
**Facial surgery procedures**	○	○	○	○	○
**Microvascular free flaps**	○	○	○	○	○
**Burn surgery procedures**	○	○	○	○	○

Do you use tranexamic acid to reduce peri- and postoperative bleeding? *

❗ Choose one of the following answers

Please choose **only one** of the following:

○ Yes

○ No

How many years have you been using tranexamic acid? *

Only answer this question if the following conditions are met:
((G01Q07.NAOK
(/index.php/questionAdministration/view/surveyid/623572/gid/6460/qid/188573) ==
"AO01"))

Choose one of the following answers
Please choose **only one** of the following:

- ◯ < 1 year
- ◯ 1-2 years
- ◯ 2-5 years
- ◯ 6-10 years
- ◯ > 10 years

General questions regarding the general use of tranexamic acid

How often do you use tranexamic acid for the prophylaxis of bleeding complications in body-contouring procedures (excluding breast surgeries)? *

Please choose the appropriate response for each item:

	< 25 %	25-49 %	50-74 %	≥ 75 %
**for aesthetic body contouring procedures**	○	○	○	○
**for postbariatric body lifting procedures**	○	○	○	○
**for liposuctions < 3 liters of lipoaspirate**	○	○	○	○
**for liposuctions > 3 liters of lipoaspirate**	○	○	○	○

How often do you use tranexamic acid for the prophylaxis of bleeding complications in breast surgery procedures (excluding oncological procedures)? *

Please choose the appropriate response for each item:

	< 25 %	25-49 %	50-74 %	≥ 75 %
**for breast augmentations with implants**	○	○	○	○
**for reduction mammaplasties**	○	○	○	○
**for mastopexies**	○	○	○	○
**for mastectomies for gynecomastia**	○	○	○	○

How often do you use tranexamic acid for the prophylaxis of bleeding complications in oncologic breast surgery procedures? *

Please choose the appropriate response for each item:

	< 25 %	25-49 %	50-74 %	≥ 75 %
**for alloplastic breast reconstructions**	○	○	○	○
**for autologous breast reconstructions**	○	○	○	○
**for mastectomies**	○	○	○	○
**for breast-conserving surgeries**	○	○	○	○

How often do you use tranexamic acid for the prophylaxis of bleeding complications in facial procedures? *

Please choose the appropriate response for each item:

	< 25 %	25-49 %	50-74 %	≥ 75 %
**for facelifts**	○	○	○	○
**for periorbital surgery**	○	○	○	○
**for rhinoplasties**	○	○	○	○

How often do you use tranexamic acid for the prophylaxis of bleeding complications in microvascular free flap surgery? *

Please choose the appropriate response for each item:

	< 25 %	25-49 %	50-74 %	≥ 75 %
**Microvascular free flaps**	○	○	○	○

How often do you use tranexamic acid for the prophylaxis of bleeding complications in acute burn surgery procedures (2b-3 degree burns)? *

Please choose the appropriate response for each item:

	< 25 %	25-49 %	50-74 %	≥ 75 %
**for 2b-3 degree burns <10% total body surface area**	○	○	○	○
**for 2b-3 degree burns 10-30% total body surface area**	○	○	○	○
**for 2b-3 degree burns >30% total body surface area**	○	○	○	○

What factors do you consider in determining the indication for administration of tranexamid acid? *

❗ Check all that apply

Please choose **all** that apply:

- [ ] Type of surgery
- [ ] Size of wound area
- [ ] Intraoperative blood loss
- [ ] Anticoagulation or coagulopathies
- [ ] Kidney function
- [ ] Liver function
- [ ] Preoperative hemoglobin level
- [ ] Known oncologic history/condition
- [ ] Patient's age
- [ ] Primary or revision procedure
- [ ] Other: ____________

What administration method for tranexamic acid do you prefer? *

❗ Choose one of the following answers

Please choose **only one** of the following:

- ○ Systemic
- ○ Topical
- ○ Combined systemic and topical

Have you observed an increase in thromboembolic events due to the administration of tranexamic acid? *

Choose one of the following answers
Please choose **only one** of the following:

- ◯ Yes
- ◯ No

With which form of application have you seen an increase in thromboembolic events? *

Only answer this question if the following conditions are met:
((G02Q09.NAOK (/index.php/questionAdministration/view/surveyid/623572/gid/6465/qid/188982) == "AO01"))

Choose one of the following answers
Please choose **only one** of the following:

- ◯ with systemic administration
- ◯ with topical administration
- ◯ with systemic and topical administration

Questions regarding the intravenous application of tranexamic acid

In which of the following procedures do you indicate the intravenous administration of tranexamic acid? *

❗ Check all that apply

Please choose **all** that apply:

- ☐ Upper/lower extremity body-lifting surgeries
- ☐ Abdominoplasties
- ☐ Liposuctions < 3 liters of lipoaspirate
- ☐ Liposuctions > 3 liters of lipoaspirate
- ☐ Circumferential body lifts
- ☐ Breast augmentations with implants (first augmentation)
- ☐ Breast re-augmentations (e.g. after capsular fibrosis)
- ☐ Reduction mammaplasties
- ☐ Mastopexies
- ☐ Mastectomies for gynecomastia
- ☐ Autologous breast reconstructions
- ☐ Alloplastic breast reconstructions
- ☐ Mastectomies
- ☐ Breast-conserving surgeries
- ☐ Facelifts
- ☐ Rhinoplasties
- ☐ Periorbital Surgery
- ☐ Microvascular free flaps
- ☐ 2b-3 degree burns < 10% total body surface area
- ☐ 2b-3 degree burns 10-30% total body surface area
- ☐ 2b-3 degree burns > 30% total body surface area
- ☐ Other: ____________

What dose in **mg/kg bodyweight (BW)** do you use for intravenous administration of tranexamic acid? *

Choose one of the following answers

Please choose **only one** of the following:

- ◯ <5 mg/kg BW
- ◯ 5-9 mg/kg BW
- ◯ 10-14 mg/kg BW
- ◯ ≥15 mg/kg BW

In what dilution do you administer tranexamic acid intravenously? *

Choose one of the following answers

Please choose **only one** of the following:

- ◯ Undiluted (bolus)
- ◯ Short-term infusion (e.g. 50-100 ml)
- ◯ Infusion (e.g. 500-1000 ml)
- ◯ Other ______

When do you administer tranexamic acid? *

❗ Check all that apply

Please choose **all** that apply:

- ☐ >60 minutes before incision
- ☐ 30-60 minutes before incision
- ☐ <30 minutes before incision
- ☐ At incision
- ☐ Intraoperatively during hemostasis
- ☐ Intraoperatively during suturing
- ☐ Postoperatively
- ☐ Other: ____________

How often do you administer tranexamic acid? *

❗ Choose one of the following answers

Please choose **only one** of the following:

- ○ Once
- ○ Multiple times
- ○ Individually once or multiple times

Depending on which criteria do you decide wheter do administer tranexamic acid once or several times? *

Only answer this question if the following conditions are met:
((G03Q05.NAOK (/index.php/questionAdministration/view/surveyid/623572/gid/6466/qid/189111) == "AO03"))

Check all that apply

Please choose **all** that apply:

- [ ] Depending on the individual bleeding risk
- [ ] Depending on the intraoperative blood loss
- [ ] Depending on the postoperative blood loss through drains
- [ ] Depending on the presence of postoperative hematomas
- [ ] Other: ____________

For how long do you administer tranexamic acid intravenously for multiple doses? *

Only answer this question if the following conditions are met:
((G03Q05.NAOK (/index.php/questionAdministration/view/surveyid/623572/gid/6466/qid/189111) == "AO02" or G03Q05.NAOK (/index.php/questionAdministration/view/surveyid/623572/gid/6466/qid/189111) == "AO03"))

Choose one of the following answers

Please choose **only one** of the following:

- ○ <24 hours
- ○ 24-72 hours
- ○ >72 hours

At what intervals do you administer the doses of tranexamic acid for multiple doses? *

Only answer this question if the following conditions are met:
((G03Q05.NAOK (/index.php/questionAdministration/view/surveyid/623572/gid/6466/qid/189111) == "AO02" or G03Q05.NAOK (/index.php/questionAdministration/view/surveyid/623572/gid/6466/qid/189111) == "AO03"))

Choose one of the following answers
Please choose **only one** of the following:

- Every 2-4 hours
- Every 4-6 hours
- Every 6-8 hours
- Every 12 hours
- Every 24 hours
- Individually based on patient's bleeding risk and blood loss
- Other

Questions regarding the topical application of tranexamic acid

In which of the following procedures do you indicate the topical application of tranexamic acid? *

❶ Check all that apply

Please choose **all** that apply:

- [ ] Upper/lower extremity body-lifting surgeries
- [ ] Abdominoplasties
- [ ] Liposuctions < 3 liters of lipoaspirate
- [ ] Liposuctions > 3 liters of lipoaspirate
- [ ] Circumferential body lifts
- [ ] Breast augmentations with implants (first augmentation)
- [ ] Breast re-augmentations (e.g. after capsular fibrosis)
- [ ] Reduction mammaplasties
- [ ] Mastopexies
- [ ] Mastectomies for gynecomastia
- [ ] Autologous breast reconstructions
- [ ] Alloplastic breast reconstructions
- [ ] Mastectomies
- [ ] Breast-conserving surgeries
- [ ] Facelifts
- [ ] Rhinoplasties
- [ ] Periorbital Surgery
- [ ] Microvascular free flaps
- [ ] 2b-3 degree burns < 10% total body surface area
- [ ] 2b-3 degree burns 10-30% total body surface area
- [ ] 2b-3 degree burns > 30% total body surface area
- [ ] Other: ____________

Depending on which criteria do you decide on the total amount of tranexamic acid for topical application? *

Check all that apply

Please choose **all** that apply:

- [ ] Body weight
- [ ] Wound surface area
- [ ] Other:

What dilution do you use for the topical application of tranexamic acid (except for liposuctions)? *

Choose one of the following answers

Please choose **only one** of the following:

- ○ 100 mg/ml (undiluted)
- ○ 50-99 mg/ml (diluted)
- ○ 25-49 mg/ml (diluted)
- ○ 10-24 mg/ml (diluted)
- ○ <10 mg/ml (diluted)

When do you apply tranexamic acid topically? *

Choose one of the following answers
Please choose **only one** of the following:

- ◯ During hemostasis
- ◯ During suturing
- ◯ Postoperatively (e.g. through a drain or by injection)
- ◯ Other ______

How do you apply tranexamic acid topically? *

Choose one of the following answers
Please choose **only one** of the following:

- ◯ Application of powder
- ◯ Through irrigation
- ◯ Through soaked compresses/abdominal pads
- ◯ Other ______

What dose of tranexamic acid do you add to the tumescent solution for liposuctions? *

Choose one of the following answers
Please choose **only one** of the following:

- ◯ <100 mg/L (<0,1 mg/ml)
- ◯ 100-240 mg/L (0,1-0,24 mg/ml)
- ◯ 250-490 mg/L (0,25-0,49 mg/ml)
- ◯ 500-990 mg/L (0,5-0,99 mg/ml)
- ◯ >1000 mg/L (>1 mg/ml)
- ◯ I do not use tranexamic acid for liposuctions

Further questions

How would you rate the benefit of using tranexamid acid to reduce/prevent postoperative bleeding complications? *

Only answer this question if the following conditions are met:
((G01Q07.NAOK (/index.php/questionAdministration/view/surveyid/623572/gid/6460/qid/188573) == "AO01"))

Choose one of the following answers
Please choose **only one** of the following:

- ◯ Very useful
- ◯ Useful
- ◯ Neutral
- ◯ Not very useful
- ◯ Not useful

Why do you not use tranexamic acid for the prophylaxis of bleeding and bleeding compliactions in plastic surgery procedures?

Only answer this question if the following conditions are met:
((G01Q07.NAOK (/index.php/questionAdministration/view/surveyid/623572/gid/6460/qid/188573) == "AO02"))

❗ Check all that apply
Please choose **all** that apply:

- [ ] I don't see any benefit
- [ ] It has a high risk of complications
- [ ] There is no sufficient evidence
- [ ] Lack of experience
- [ ] I have not considered so far
- [ ] It is not approved in my country
- [ ] Other: ______

What other methods do you use to reduce/prevent postoperative bleeding complications? *

❗ Check all that apply
Please choose **all** that apply:

- [ ] Elevation of systolic blood pressure during hemostasis
- [ ] Postoperative compression
- [ ] Local application of hemostatic agents (e.g. Tabotamp®)
- [ ] Local application of adrenaline to the wound surface
- [ ] Addition of adrenaline to local anesthesia or tumescent solution
- [ ] Other: ______

Which of the following complications have you already observed when using tranexamic acid? *

Only answer this question if the following conditions are met:
((G01Q07.NAOK (/index.php/questionAdministration/view/surveyid/623572/gid/6460/qid/188573) == "AO01"))

Check all that apply
Please choose **all** that apply:

- [ ] None
- [ ] Allergic reaction
- [ ] Thromboembolic event
- [ ] Visual impairment
- [ ] Seizure
- [ ] Other: ____________

What are your experiences with the anesthesiologists regarding their feedback on the indication for using tranexamic acid? *

Only answer this question if the following conditions are met:
((G01Q07.NAOK (/index.php/questionAdministration/view/surveyid/623572/gid/6460/qid/188573) == "AO01"))

❗ Choose one of the following answers
Please choose **only one** of the following:

- ◯ Anesthesiologists agree with the indication and consider it appropriate
- ◯ Although anesthesiologists do not agree with the indication, they still administer tranexamic acid
- ◯ Anesthesiologists decline the administration of tranexamic acid
- ◯ Other [ ]

Thank you for participating in our survey!

Your answers have been saved. You can now close the window.

Submit your survey.
Thank you for completing this survey.
